# Metathetical Redox Reaction of (Diacetoxyiodo)arenes and Iodoarenes

**DOI:** 10.3390/molecules201219874

**Published:** 2015-12-17

**Authors:** Antoine Jobin-Des Lauriers, Claude Y. Legault

**Affiliations:** Centre in Green Chemistry and Catalysis, Department of Chemistry, University of Sherbrooke, 2500 boul. de l’Université, Sherbrooke, QC J1K 2R1, Canada; Antoine.Jobin-Des.Lauriers@USherbrooke.ca

**Keywords:** hypervalent iodine, (diacetoxyiodo)arenes, metathesis

## Abstract

The oxidation of iodoarenes is central to the field of hypervalent iodine chemistry. It was found that the metathetical redox reaction between (diacetoxyiodo)arenes and iodoarenes is possible in the presence of a catalytic amount of Lewis acid. This discovery opens a new strategy to access (diacetoxyiodo)arenes. A computational study is provided to rationalize the results observed.

## 1. Introduction

**Scheme 1:**
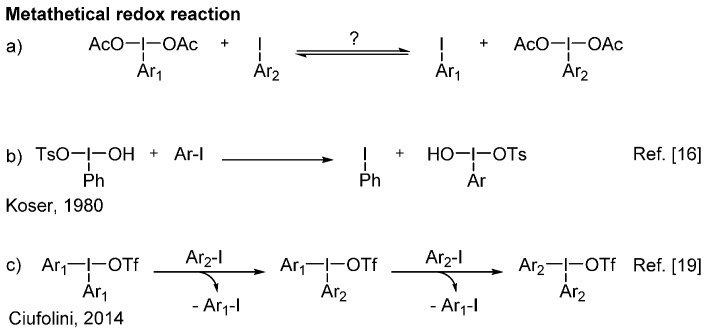
Concept and examples of metathetical redox reactions of iodanes and iodoarenes.

In the recent years, the development of hypervalent iodine-mediated synthetic transformations has been receiving growing attention [1,2,3,4,5]. This is not surprising considering the importance of improving oxidative reactions and reducing their environmental impact. Hypervalent iodine reagents are a great alternative to toxic heavy metals often used to effect similar transformations [6,7,8,9,10]. Among the plethora of iodine(III) compounds [11], it is undeniable that the (diacetoxyiodo)arenes remain the most common and used ones [12]. They are usually stable solids and can serve as precursors to numerous other iodanes, such as other (diacyloxyiodo)arenes and [hydroxy(tosyloxy)iodo]arenes [13,14]. Access to (diacetoxyiodo)arenes is, of course, usually accomplished by the oxidation of the corresponding iodoarene substrate. The traditional method involves peracetic acid oxidation in acetic acid [15]. One unexploited strategy to access these specific reagents is the metathetical redox reaction between (diacetoxyiodo)arenes and related iodoarenes (Scheme 1a).

While it is widely accepted that the ligands on iodane species are typically easily exchanged, the interconversion of oxidation states between λ^3^-iodane and iodoarenes is much rarer. The first example of such a metathetical redox reaction was reported by Koser *et al.*, using [hydroxy(tosyloxy)-iodo]benzene (HTIB) as the oxidant, enabling transfer to a variety of iodoarenes (Scheme 1b) [16]. Two applications of this initial method were subsequently reported, including one example using [bis(trifluoroacetoxy)iodo]benzene (BTI) [17,18]. More recently, Ciufolini *et al.* have reported the iodonium salt metathesis reaction with iodoarenes (Scheme 1c) [19].

To the best of our knowledge, the metathetical redox reaction between (diacetoxyiodo)arenes and iodoarenes has never been reported. Expanding the scope of iodane metathesis to these commonly used reagents would be of great interest as an alternative synthetic method. Herein we report a protocol to achieve such a metathesis of (diacetoxyiodo)benzene and iodoarenes. We also provide a computational study to determine the origin of the observed reactivity.

## 2. Results and Discussion

**Scheme 2:**
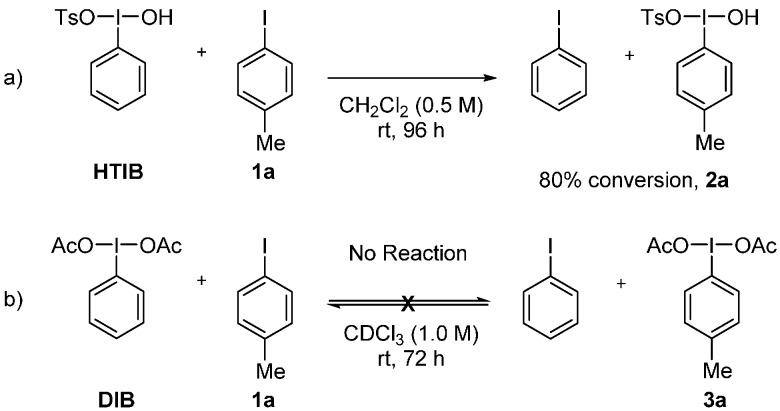
Evaluation of the reactivity of DIB toward the metathetical redox process.

The reactivity of HTIB and (diacetoxyiodo)benzene (DIB) toward iodoarenes was evaluated, using conditions analogous to Koser’s successful iodane metathesis with HTIB [16]. We submitted *p*-methyliodobenzene and *p*-bromoiodobenzene to iodane metathesis with HTIB (Scheme 2a) and DIB at room temperature (Scheme 2b). In accordance with the results reported by Koser, equilibrium is achieved for the reaction with HTIB within 96 h. In contrast, no metathesis was observed after 72 h with DIB.

**Scheme 3:**
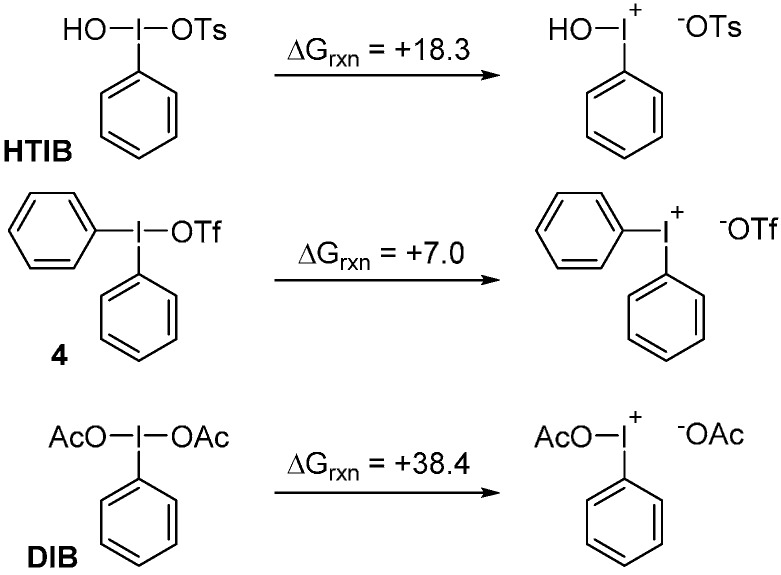
Energetics of ligand dissociation on DIB, **4**, and HTIB (energies reported in kcal/mol).

These results demonstrate the very different reactivity profiles of DIB and HTIB. The origin of this reactivity dichotomy was investigated. In a recent computational study, we have suggested that reaction with HTIB would involve, at room temperature, iodonium intermediates, through a ligand dissociation mechanism [20]. Due to the large difference in effective electronegativity between the hydroxy and tosyloxy groups on HTIB, ionization in solution is achievable at room temperature [21,22]. In contrast, (diacetoxyiodo)arenes have identical ligands involved in the three-center four-electron bonding, and thus dissociation is expected to be difficult [23]. We have evaluated the energetic of dissociation for HTIB, DIB, and diphenyliodonium salt **4** (see the computational details in Section 4), the results are illustrated in Scheme 3.

As suspected, the dissociation of the tosyloxy group is calculated to be endergonic, but feasible. Dissociation of the triflate anion in the diphenyliodonium salt **4** is predicted to be even more facile at room temperature. In contrast, the calculations suggest that dissociation of an acetoxy group on DIB at room temperature would be too difficult. 

**Scheme 4:**
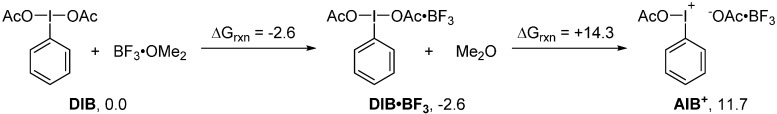
Energetics of ligand dissociation on DIB with BF_3_·OMe_2_ (energies reported in kcal/mol).

The dissociation energies seem to be correlated to the intrinsic reactivity of these iodanes toward metathesis. We postulated that the key to the metathetical redox process might occur by the formation of an iodonium intermediate. If it is the case, this could be achieved with DIB by promoting the dissociation of an acetoxy group through the use of a Lewis acid. We elected to use BF_3_·OEt_2_, as it was shown in numerous methodologies to be a proficient and compatible Lewis acid with DIB [24,25]. DFT Calculations, using BF_3_·OMe_2_ as a model, suggest that this Lewis acid would permit the formation of an iodonium intermediate at room temperature, as illustrated in Scheme 4.

With this in mind, we tested the iodane metathesis between DIB and *p*-bromoiodobenzene (**1b**) in the presence of 5 mol % of BF_3_·OEt_2_ (Equation (1)). To our delight, the metathetical redox process proceeded cleanly to attain equilibrium within 67 h. The calculated free energy of reaction was predicted to be +0.5 kcal/mol, which is in good agreement with the experimentally measured value of +0.24 kcal/mol. The reaction did also proceed with *p*-methyliodobenzene (**1a**), but formation of a suspension pointed to a Friedel-Crafts side reaction of **1a** with activated DIB, and was not investigated further.

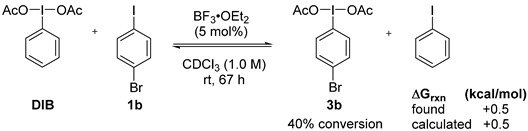
(1)

With these promising results in hand, the reaction mechanism was investigated using DFT calculations (see computational details in Section 4). A single electron transfer (SET) redox process between the iodonium intermediate and the iodoarene was considered (Equation (2)). The transfer was found to be energetically too costly to occur readily at room temperature. In accordance with the calculations, it was found that light had no effect on the rate of reaction in the conditions described in Equation (1), further suggesting that the process does not involve a SET.

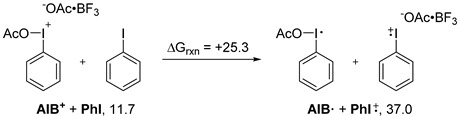
(2)

**Scheme 5:**
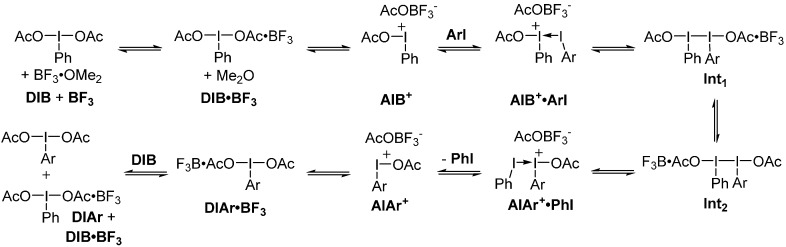
Reaction mechanism evaluated using DFT calculations (Ar = Ph for simplicity).

An ionic mechanism was thus considered. The intermediates evaluated are illustrated in Scheme 5. This mechanism has similarities to the one proposed by Ciufolini *et al.* for diaryliodonium salt metathesis [19], with the exception of the transferred group being easily dissociated. For the sake of simplicity, the aryl (Ar) group in the intermediates is a simple phenyl group and the initial iodane is DIB. The transition states leading to the different intermediates described were not studied, as it was assumed that these processes of association and dissociation do not require large activation energies.

**Scheme 6:**
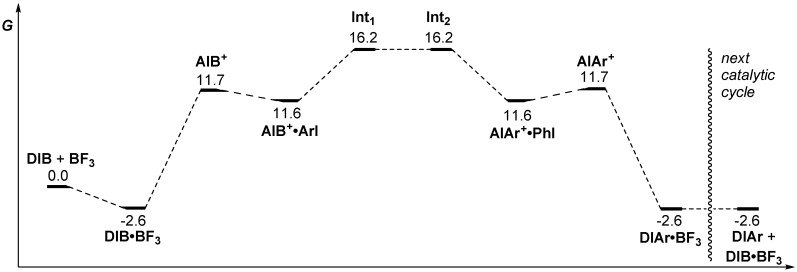
Energy diagram of the proposed mechanism (energies reported in kcal/mol).

The energetic properties of all these intermediates were calculated and are illustrated in an energy diagram (Scheme 6). As described in Scheme 6, formation of intermediate **AIB^+^**, following complexation by BF_3_·OMe_2_, was found to be an endergonic but feasible process at room temperature. At this point, the iodonium intermediate can act as a Lewis acid toward an iodoarene (in this case iodobenzene) to form adduct **AIB^+^·ArI**. Association of the AcOBF_3_^−^ counterion led to **Int_1_**. By internal Lewis acid migration, **Int_2_** would be accessed. This intermediate can then lead to **AIAr^+^·PhI** adduct, which can liberate iodobenzene and finally provide, by transfer of BF_3_ to another DIB molecule, the newly formed (diacetoxyiodo)arene (**DIAr**). The calculations thus suggest an ionic mechanism relying on the asymmetry or activation of the iodane moiety to initiate the metathetical redox process, through a key iodonium intermediate. 

Passage through a non-symmetric iodane intermediate could explain the metathesis reported by Koser *et al.*, in which iodoarene **5** is converted to its corresponding cyclic iodane **6** by the action of BTI, without the need of a catalyst (Equation (3)) [17].

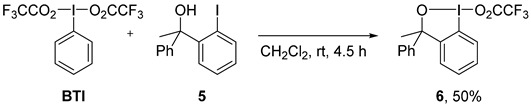
(3)

**Scheme 7:**
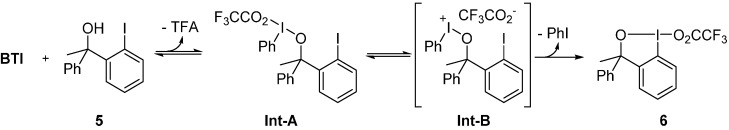
Proposed intermediate to rationalize reactivity of BTI toward **5**.

In this reaction, rapid ligand exchange would lead to intermediate **Int-A** (Scheme 7). Due to the large difference in effective electronegativity between the alkoxy and trifluoroacetoxy groups, dissociation of the latter to furnish the iodonium intermediate **Int-B** is conceivable. Furthermore, the fact that **Int-B** binds together both I(I) and I(III) partners might facilitate the metathetical redox process.

**Scheme 8:**
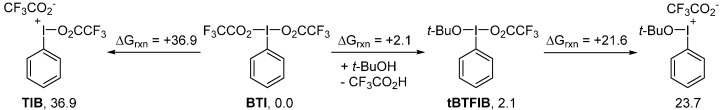
Alcohol-promoted formation of an iodonium intermediate.

Calculations using *t*-BuOH as a model alcohol support this proposal, as described by the energetic values illustrated in Scheme 8. While dissociation of a trifluoroacetate group on the symmetric BTI was calculated to be too costly to occur at room temperature. In contrast, due to the lower effective electronegativity of the *t*-butoxy compared to the trifluoroacetoxy group, the dissociation on the non-symmetric tBTFIB is facilitated. 

**Scheme 9:**
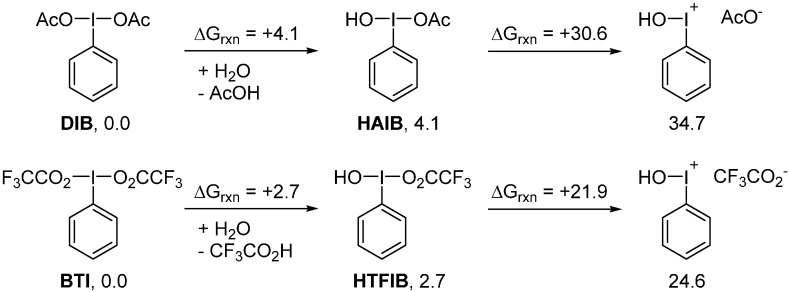
Water-promoted formation of iodonium intermediates.

This alternative activation mechanism raises an important point concerning the presence of water in these reactions. As a protic solvent similar to an alcohol, water might act as an activator to promote acyloxy group dissociation. The energetic values of water exchange and possible acyloxy dissociation were computed for both DIB and BTI; the results are illustrated in Scheme 9. In the case of DIB, even the intermediate species [hydroxy(acetyloxy)iodo]benzene (HAIB) would not permit dissociation of the acetyloxy group to form the iodonium intermediate at room temperature. In contrast, the calculations suggest that dissociation of the trifluoroacetoxy group on the analogous [hydroxy(trifluoroacetoxy)iodo]benzene (HTFIB) intermediate might be possible at room temperature. In essence, even if BTI is found to be more stable than HTFIB, the latter would possess reactivity similar to Koser’s reagent (HTIB). The main difference between DIB, BTI, and HTIB can simply be explained by the better nucleofugality of TsO^−^ and CF_3_CO_2_^−^ *vs.* AcO^−^.

**Scheme 10:**
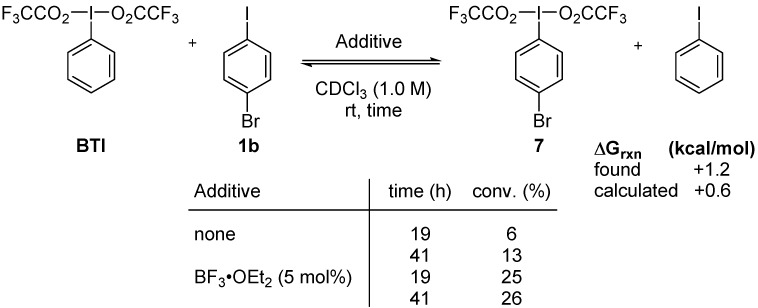
Evaluation of BTI toward uncatalyzed and catalyzed iodane metathesis.

These calculations support the non-reactivity observed for DIB even in non-anhydrous solvents. In contrast, we could expect reactivity from BTI even at room temperature under non-anhydrous conditions. To test this hypothesis, BTI was let to react with *p*-bromoiodobenzene at room temperature without strict exclusion of water. For comparison, a similar experiment involving 5 mol % of BF_3_·OEt_2_ was also performed. The results are illustrated in Scheme 10.

The experimental outcome of the iodane metathesis reactions with BTI is in agreement with the calculation results. Metathesis occurs slowly in the absence of a Lewis acid; equilibrium could not be achieved, even after 41 h. As with DIB, the Lewis acid has a drastic effect on the rate of metathesis, as equilibrium is attained within 19 h. Again, the calculated free energy of reaction is in good agreement with the experimentally observed conversion (Scheme 10).

**Scheme 11:**
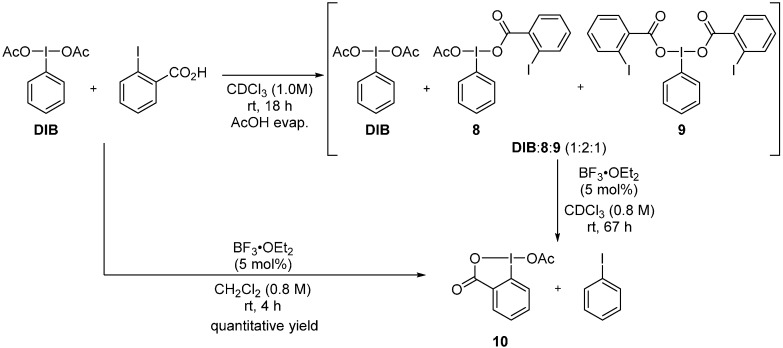
Ligand exchange between DIB and *o*-I-BzOH followed by iodane metathesis.

As the iodane metathesis reactions are under thermodynamic control, we selected a substrate that would favor a complete metathetical redox process. We performed the iodane metathesis using DIB and *o*-iodobenzoic acid (*o*-I-BzOH) as the iodoarene (Scheme 11). The thermodynamic drive (*i.e.*, entropic gain) of the reaction was predicted to lead to a complete conversion (ΔG_rxn_ = −11.8 kcal/mol). Additionally, *o*-I-BzOH enables the evaluation of the proximity effect of the iodine(I) and iodine(III) partners on the rate of the metathetical redox reaction. Indeed, *o*-I-BzOH can serve as an acyloxy ligand. We thus performed AcO/*o*-I-BzO exchange on DIB in the absence of a Lewis acid. Treatment of an equimolar mixture of DIB and *o*-I-BzOH in chloroform, followed by AcOH removal through hexane azeotrope, resulted in a clean mixture of DIB, **8**, and **9** in a 1:2:1 ratio, indicative of an almost statistical equilibrium. It is important to note that no iodobenzene was observed at this point. The proximity effect of the I(I) and I(III) partners did not facilitate in any way the metathetical redox process in the absence of a Lewis acid. Treatment of this statistical mixture of [bis(acyloxy)iodo]benzenes with a catalytic quantity of BF_3_·OEt_2_ resulted in the smooth and clean conversion to the single cyclic iodane **10** and iodobenzene. The same efficient metathetical redox process could be done in one-pot by treating an equimolar quantity of DIB and *o*-I-BzOH with 5 mol % of BF_3_·OEt_2_ for 4 h in dichloromethane. Compound **10** could be obtained in essentially quantitative yield by simple removal of PhI by trituration with hexane.

**Scheme 12:**
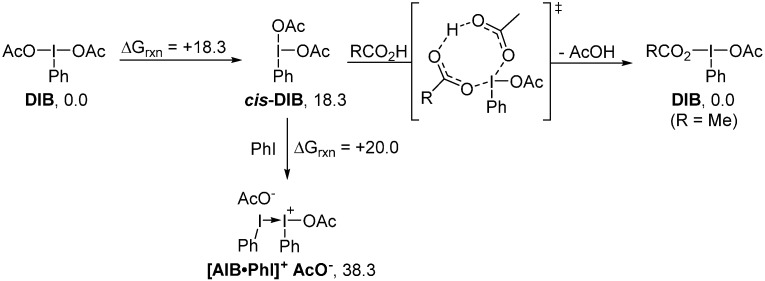
Plausible mechanism for ligand exchange (energies reported in kcal/mol).

There is a need to reconcile the fact that ligand exchange on DIB is facile at room temperature without the need for any activation, while the metathetical redox process is not possible. The nature of the exchange processes needs to be considered. While the calculations suggest a mandatory dissociative process through an iodonium intermediate to achieve metathesis, an associative mechanism could still be considered for ligand exchange (Scheme 12). It was calculated that *cis*-DIB, a T-shaped isomer of DIB in which both acetoxy ligands are *cis* to each other, would be energetically accessible at room temperature. This intermediate neutral iodane would react readily with a carboxylic acid (AcOH was used for simplicity), through proton transfer, to afford in return DIB in its most stable conformation, bearing its two most electron withdrawing groups in *trans* relation. Addition of iodobenzene on *cis*-DIB cannot occur readily, as it is not a strong enough donor to stabilize expulsion of the acetoxy group.

In summary, we have demonstrated that, with the simple action of a Lewis acid, it is possible to achieve the metathetical redox reaction of (diacetoxyiodo)arenes and iodoarenes. Both the experimental results and computational insights draw a clearer picture of what is needed for these metathetical redox reactions to proceed. The results presented herein open the way for further exploration.

## 3. Experimental Section 

### 3.1. General Information

All glassware was stored in the oven and/or was flame dried prior to use under an inert atmosphere of gas. CDCl_3_ was dried over anhydrous K_2_CO_3_ but not thoroughly dried. Analytical thin-layer chromatography (TLC) was performed on precoated, glass-backed silica gel (Merck 60 F_254_). Visualization of the developed chromatogram was performed by UV absorbance, aqueous cerium molybdate, ethanolic phosphomolybdic acid, iodine, or aqueous potassium permanganate. Nuclear magnetic resonance spectra (^1^H, ^13^C, DEPT) were recorded either on an Avance III HD 300 (Bruker, Billerica, MA, USA) or Mercury+ 400 (Agilent Technology, Santa Clara, CA, USA) spectrometers. Chemical shifts for ^1^H-NMR spectra are recorded in parts per million from tetramethylsilane with the solvent resonance as the internal standard. Data are reported as follows: chemical shift, multiplicity (s = singlet, d = doublet, t = triplet, q = quartet, qn = quintet, sext = sextuplet, m = multiplet and br = broad), coupling constant in Hz, integration. Chemical shifts for ^13^C-NMR spectra are recorded in parts per million from tetramethylsilane with the solvent resonance as the internal standard. All spectra were obtained with complete proton decoupling. When ambiguous, proton and carbon assignments were established using COSY, NOESY, HMQC and DEPT experiments. High resolution mass spectra were recorded on a Maxis ESI-Q-Tof (Bruker, Billerica, MA, USA) at the Université de Sherbrooke.

### 3.2. Metathetical Redox Reaction of HTIB and p-Methyliodobenzene

To a round-bottom flask were added [hydroxy(tosyloxy)iodo]benzene (HTIB) (333.4 mg, 0.850 mmol), *p*-methyliodobenzene (185.3 mg, 0.850 mmol) and dichloromethane (1.7 mL, 0.5 M). The white suspension was stirred at room temperature. Aliquots (5–10 μL) were taken from clear dichloromethane supernatant after allowing the suspension to settle and diluted in HPLC-grade hexanes (500–750 μL) at various moments. Conversions were determined by GC-MS by integration of signals of iodobenzene and *p*-methyliodobenzene.

### 3.3. Lewis-Acid-Catalyzed Metathetical Redox Reaction of DIB and p-Bromoiodobenzene

To a flame-dried vial equipped with a septum, under an argon atmosphere, were added DIB (372.1 mg, 1.155 mmol), *p*-bromoiodobenzene (325.6 mg, 1.155 mmol) and CDCl_3_ (442 μL). Diluted BF_3_·Et_2_O (713 μL of a freshly made solution of BF_3_·Et_2_O [100 μL BF3·Et2O in 10 mL CDCl3], 0.058 mmol) was added for a total volume of 1.16 mL of CDCl_3_ (1 M). The solution was stirred at room temperature under inert atmosphere. Conversions were determined by integration of well-defined ^1^H-NMR signals (5 s relaxation time) of the two (diacetoxyiodo)arenes present in solution. The free energies of reaction (Equation (1) and Scheme 10) were determined with the measured conversions (see Supplementary Materials for more details).

### 3.4. One-Pot Metathetical Redox Reaction of DIB and o-Iodobenzoic Acid

To a flame-dried vial, under argon, were added *o*-iodobenzoic acid (247.7 mg, 1.00 mmol), DIB (322.1 mg, 1.00 mmol) and a solution of BF_3_·Et_2_O (1.22 mL, [100 μL BF_3_·Et_2_O in 10 mL CH_2_Cl_2_], 0.012 mmol). The final reaction concentration is 0.8 M. The solution was allowed to stir at room temperature for 4 h. The solvent was evaporated and the crude residue washed twice with hexane. The crude product was dried under vacuum to afford compound **10** in quantitative yield. The spectral data is consistent with the reported one in the literature [26].

## 4. Computational Details 

The geometry optimizations were done using the Gaussian 09 software package [27] with the M06-2X [28] density functional in combination with the 6-31+G(d,p) basis set [29,30] for all atoms except iodine, for which LANL2DZdp + ECP was used [31,32]. The structures were optimized with a solvation model (SMD) for chloroform [33]. Unless otherwise stated, a fine grid density was used for numerical integration in the calculations. Conformational searches were done on all the species described throughout the mechanistic pathways. Harmonic vibrational frequencies were computed for all optimized structures to verify that they were either minima or transition states, possessing zero or one imaginary frequency, respectively. All the free energies are reported in kcal/mol and incorporate unscaled free energy corrections based on the vibrational analyses and temperature of 298 K.

## Supplementary Materials

Supplementary materials can be accessed at: http://www.mdpi.com/1420-3049/20/12/19874/s1. Cartesian coordinates, electronic and zero-point vibrational energies.
