# Supplementary material for: Metathetical Redox Reaction of (Diacetoxyiodo)arenes and Iodoarenes
Source: Molecules. 2015 Dec 17;20(12):22635–44. doi: 10.3390/molecules201219874 (PMC6332457; doi:10.3390/molecules201219874)
Supplement: Supplementary file 1 [file molecules-20-19874-s001.pdf]

# Supplementary Materials: Metathetical Redox Reaction of (Diacetoxyiodo)arenes and Iodoarenes

**Antoine Jobin-Des Lauriers and Claude Y. Legault**

## 1. Determination of Free Energies of Reaction (Equation (1) and Scheme 10)

The experimental free energies of reaction were determined based on the conversion measured at equilibrium (*i.e.*, once the conversion does not change). Based on the following reaction stoichiometry, the equilibrium constant is defined as:

$$K_{eq} = [ArI(OAc)_2][PhI]/[DIB][ArI]$$

Since the reactions are done at 1 M concentration, the equilibrium constant can be calculated as:

$$K_{eq} = [conversion][conversion]/[1 - conversion][1 - conversion]$$
$$= [conversion]^2/[1 - conversion]^2$$

Hence for Equation (1):

$$K_{eq} = [0.40\ M]^2/[0.60\ M]^2 = 0.44444$$

And for Scheme 10:

$$K_{eq} = [0.26\ M]^2/[0.74\ M]^2 = 0.12344$$

Applying

$$\Delta G_{rxn} = -RT\ ln(K_{eq})\ @\ 298K$$

Then for Equation (1),

$$\Delta G_{rxn} = +0.5\ kcal/mol$$

And for Scheme 10,

$$\Delta G_{rxn} = +1.2\ kcal/mol$$

## 2. NMR Studies Related to Ligands Exchange on DIB with *o*-Iodobenzoic Acid

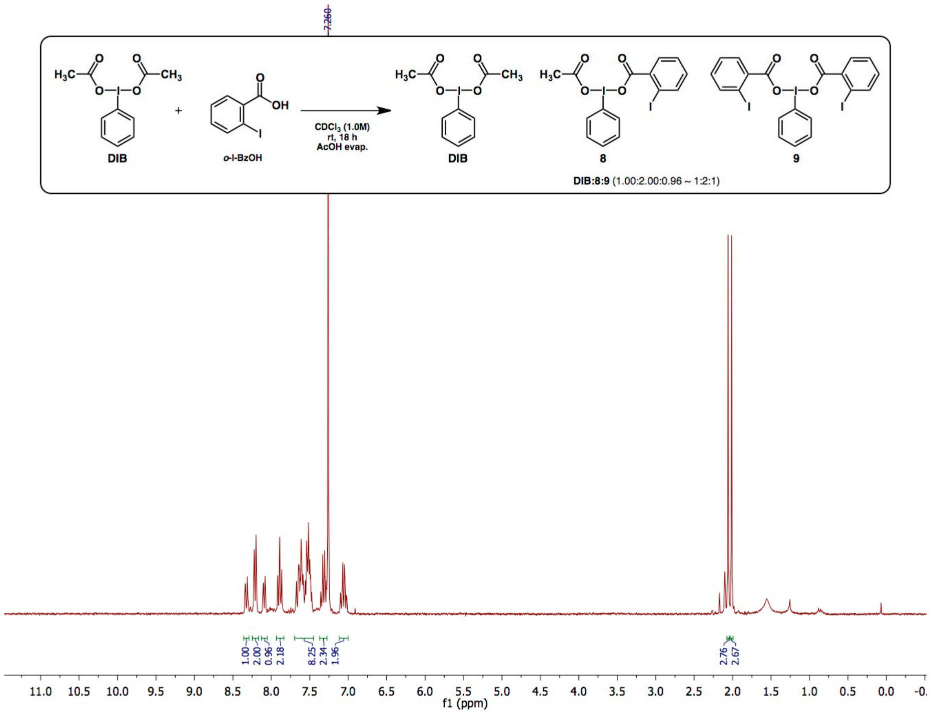

**Figure S1**. $^{1}$H NMR analysis (integration) of ligand exchange on DIB with o-I-BzOH (Scheme 11).

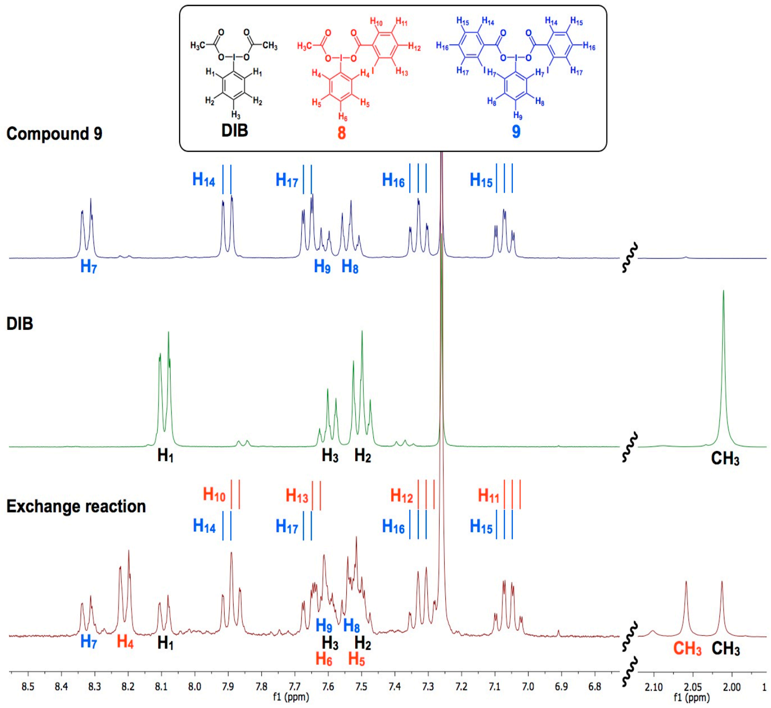

**Figure S2**. $^{1}$H NMR analysis (assignation) of ligand exchange on DIB with *o*-I-BzOH (Scheme 11).

## 3. Optimized Structures

**$AcO^-$ anion**

| | | | |
|---|---|---|---|
| H | -3.080957 | -0.767315 | -2.677382 |
| H | -2.877660 | 0.167601 | -4.181137 |
| O | -0.366802 | 0.079833 | -3.926995 |
| C | -1.097068 | 0.076435 | -2.902375 |
| C | -2.619757 | 0.121458 | -3.120823 |
| O | -0.711994 | 0.038178 | -1.703650 |
| H | -3.039916 | 0.992995 | -2.608105 |

SCF energy: -228.524504722
opt@M06-2X(SMD,CHCl3)/6-31+G(d,p)
No imaginary frequency
Zero-point correction: 0.049037
Enthalpy correction: 0.054345
Free energy correction: 0.021646

**$AcOBF_3^-$ anion**

| | | | |
|---|---|---|---|
| B | -0.093880 | 0.128200 | 0.928232 |
| F | -0.087741 | -0.332726 | -0.401742 |
| F | 1.183617 | 0.610276 | 1.253363 |
| F | -1.043157 | 1.154777 | 1.055234 |
| O | -0.452520 | -1.038010 | 1.762216 |
| C | -0.543026 | -0.960297 | 3.079437 |
| O | -0.347253 | 0.054422 | 3.725647 |
| C | -0.911212 | -2.281119 | 3.706488 |
| H | -0.123243 | -3.010816 | 3.498067 |
| H | -1.836165 | -2.658132 | 3.262077 |
| H | -1.032420 | -2.167475 | 4.783380 |

SCF energy: -553.077984770
opt@M06-2X(SMD,CHCl3)/6-31+G(d,p)
No imaginary frequency
Zero-point correction: 0.064085
Enthalpy correction: 0.073266
Free energy correction: 0.029939

**AcOH**

| | | | |
|---|---|---|---|
| H | -3.085679 | -0.746044 | -2.625047 |
| H | -2.833567 | 0.093600 | -4.186679 |
| O | -0.295539 | 0.184802 | -3.747905 |
| C | -1.146784 | 0.106288 | -2.888733 |
| C | -2.626866 | 0.114361 | -3.118073 |
| O | -0.837020 | 0.011087 | -1.584722 |
| H | -3.051415 | 1.019848 | -2.674898 |
| H | 0.132716 | 0.025243 | -1.494410 |

SCF energy: -229.009660313
opt@M06-2X(SMD,CHCl3)/6-31+G(d,p)
No imaginary frequency
Zero-point correction: 0.062189
Enthalpy correction: 0.067632
Free energy correction: 0.035274

**$BF_3 \cdot Me_2O$ complex**

| | | | |
|---|---|---|---|
| O | -0.041168 | -0.000008 | 1.130614 |
| B | 1.497883 | -0.000003 | 1.466597 |
| F | 1.992739 | -1.149549 | 0.895782 |
| F | 1.992728 | 1.149557 | 0.895798 |
| F | 1.598522 | -0.000012 | 2.844143 |
| C | -0.759558 | -1.204715 | 1.503493 |
| H | -0.173355 | -2.049225 | 1.149403 |
| H | -1.727014 | -1.172798 | 1.003240 |
| H | -0.877902 | -1.230516 | 2.589007 |
| C | -0.759567 | 1.204695 | 1.503489 |
| H | -0.173387 | 2.049208 | 1.149367 |
| H | -0.877884 | 1.230514 | 2.589005 |
| H | -1.727036 | 1.172753 | 1.003262 |

SCF energy: -479.470312727
opt@M06-2X(SMD,CHCl3)/6-31+G(d,p)
No imaginary frequency
Zero-point correction: 0.096099
Enthalpy correction: 0.104359
Free energy correction: 0.064672

**BTI - $PhI(O_2CCF_3)_2$**

| | | | |
|---|---|---|---|
| C | -0.002560 | -0.031030 | 0.583704 |
| C | -0.000009 | -0.054919 | 3.333067 |
| C | -0.264557 | -1.232261 | 1.237242 |
| C | 0.260435 | 1.158971 | 1.257313 |
| C | 0.262166 | 1.133658 | 2.651428 |
| C | -0.263704 | -1.231453 | 2.631549 |
| H | -0.465023 | -2.142421 | 0.681855 |
| H | 0.460382 | 2.078252 | 0.717002 |
| H | 0.466664 | 2.047028 | 3.200578 |
| H | -0.466820 | -2.154428 | 3.164953 |
| H | 0.001313 | -0.064379 | 4.418532 |
| I | -0.003328 | -0.015312 | -1.502071 |
| O | -2.130348 | 0.120412 | -1.240640 |
| C | -2.796036 | 0.128020 | -2.353275 |
| O | -2.353708 | 0.078507 | -3.480061 |
| C | -4.318098 | 0.210175 | -2.091377 |
| F | -5.003655 | 0.222977 | -3.231761 |
| F | -4.621385 | 1.321418 | -1.407801 |
| F | -4.728373 | -0.839872 | -1.367505 |
| O | 2.124707 | -0.150396 | -1.244504 |
| C | 2.788772 | -0.137311 | -2.357980 |
| O | 2.344189 | -0.079027 | -3.483480 |
| C | 4.312104 | -0.205658 | -2.099795 |
| F | 4.709922 | 0.831355 | -1.350969 |
| F | 4.996026 | -0.182022 | -3.240941 |
| F | 4.630619 | -1.329484 | -1.443863 |

SCF energy: -1294.924733930
opt@M06-2X(SMD,CHCl3)/6-31+G(d,p)+LANL2DZdp(I)
No imaginary frequency
Zero-point correction: 0.149022
Enthalpy correction: 0.170465
Free energy correction: 0.094011

**$CF_3CO_2^-$ anion**

```
F    -3.164328    -0.970882    -2.588839
F    -2.932874     0.179460    -4.400659
O    -0.324433     0.076636    -3.930719
C    -1.007673     0.074028    -2.894344
C    -2.556967     0.119376    -3.111814
O    -0.693900     0.039560    -1.691254
F    -3.113979     1.191007    -2.502838
```

```
SCF energy: -526.186833409
opt@M06-2X(SMD,CHCl3)/6-31+G(d,p)
    No imaginary frequency
    Zero-point correction:      0.026575
    Enthalpy correction:        0.033496
    Free energy correction:     -0.004949
```

**$CF_3CO_2H$**

```
F    -3.165455    -0.944878    -2.535348
F    -2.868847     0.096870    -4.409474
O    -0.272930     0.170603    -3.781466
C    -1.063812     0.102905    -2.883089
C    -2.589809     0.115510    -3.111097
O    -0.810125     0.021140    -1.589065
F    -3.127801     1.218256    -2.576069
H     0.154626     0.028779    -1.434860
```

```
SCF energy: -526.641257292
opt@M06-2X(SMD,CHCl3)/6-31+G(d,p)
    No imaginary frequency
    Zero-point correction:      0.039540
    Enthalpy correction:        0.046658
    Free energy correction:     0.008506
```

***cis*-DIB**

```
C     0.111789    -0.099297     0.285352
C     0.107039    -0.146939     3.062991
C     0.331088    -1.306767     0.954274
C    -0.114844     1.083411     0.994371
C    -0.116880     1.054389     2.388779
C     0.329952    -1.325185     2.348947
H     0.501606    -2.222897     0.395402
H    -0.287876     2.017012     0.466387
H    -0.292713     1.969017     2.946722
H     0.500836    -2.259036     2.875682
H     0.105185    -0.165367     4.148700
I     0.079285    -0.077563    -1.833429
O     2.017556     0.356601    -1.712922
C     2.888359    -0.669150    -1.873608
O     2.540111    -1.820165    -1.973296
C     4.299624    -0.159715    -1.908532
H     4.436666     0.420143    -2.825664
H     4.987619    -1.003938    -1.895698
H     4.484153     0.499635    -1.058026
C    -0.097324    -0.139726    -4.819308
O     0.880825     0.032299    -3.987323
C     0.293671    -0.144471    -6.279682
H     0.812187     0.786165    -6.525546
H    -0.589235    -0.255767    -6.909601
H     0.986995    -0.969504    -6.466466
O    -1.271837    -0.292277    -4.456892
```

```
SCF energy: -699.620843592
opt@M06-2X(SMD,CHCl3)/6-31+G(d,p)+LANL2DZdp(I)
    No imaginary frequency
    Zero-point correction:      0.193985
    Enthalpy correction:        0.211793
    Free energy correction:     0.145240
```

**Cyclic iodane (10)**

| | | | |
|---|---|---|---|
| C | 0.003138 | 0.060070 | 1.016389 |
| C | 0.033376 | 0.090982 | 3.771728 |
| C | -0.023012 | -1.144226 | 1.708452 |
| C | 0.044113 | 1.291799 | 1.655347 |
| C | 0.058564 | 1.287765 | 3.051790 |
| C | -0.007046 | -1.128229 | 3.102738 |
| H | 0.064071 | 2.219179 | 1.097362 |
| H | 0.089689 | 2.236261 | 3.578656 |
| H | -0.028181 | -2.073179 | 3.636613 |
| H | 0.044906 | 0.113073 | 4.856483 |
| C | -0.070745 | -2.419896 | 0.926553 |
| O | -0.091292 | -3.518366 | 1.445404 |
| O | -0.089813 | -2.230763 | -0.381068 |
| I | -0.031050 | -0.235578 | -1.058435 |
| O | 0.038111 | 1.860067 | -1.174655 |
| C | -0.007608 | 2.287449 | -2.428219 |
| O | -0.070656 | 1.510615 | -3.369897 |
| C | 0.019391 | 3.783781 | -2.556196 |
| H | 0.907025 | 4.179271 | -2.056423 |
| H | -0.860199 | 4.203689 | -2.060889 |
| H | 0.023236 | 4.063631 | -3.608813 |

SCF energy: -659.157161476
opt@M06-2X(SMD,CHCl3)/6-31+G(d,p)+LANL2DZdp(I)
No imaginary frequency
Zero-point correction: 0.146013
Enthalpy correction: 0.160221
Free energy correction: 0.104156

**DIB - PhI(OAc)$_2$**

| | | | |
|---|---|---|---|
| C | -0.002209 | -0.032694 | 0.617641 |
| C | 0.000391 | -0.056491 | 3.377736 |
| C | -0.050133 | -1.257577 | 1.278908 |
| C | 0.047079 | 1.180808 | 1.299738 |
| C | 0.047186 | 1.158750 | 2.694441 |
| C | -0.047645 | -1.259766 | 2.673814 |
| H | -0.088613 | -2.189417 | 0.724177 |
| H | 0.084562 | 2.121883 | 0.760875 |
| H | 0.084992 | 2.094468 | 3.243081 |
| H | -0.084438 | -2.204925 | 3.206111 |
| H | 0.001380 | -0.065821 | 4.463331 |
| I | -0.004295 | -0.015780 | -1.472950 |
| O | -2.116070 | 0.139942 | -1.178728 |
| C | -2.769312 | 0.200016 | -2.319970 |
| O | -2.193417 | 0.177506 | -3.402605 |
| C | -4.263538 | 0.291167 | -2.165819 |
| H | -4.734249 | 0.379952 | -3.144364 |
| H | -4.517188 | 1.154981 | -1.546305 |
| H | -4.628686 | -0.604525 | -1.656072 |
| O | 2.108407 | -0.173497 | -1.185080 |
| C | 2.761844 | -0.204779 | -2.327323 |
| O | 2.185939 | -0.165319 | -3.409436 |
| C | 4.256926 | -0.284804 | -2.174856 |
| H | 4.613479 | 0.605129 | -1.649143 |
| H | 4.728200 | -0.351263 | -3.154893 |
| H | 4.519104 | -1.157142 | -1.571108 |

SCF energy: -699.650726352
opt@M06-2X(SMD,CHCl3)/6-31+G(d,p)+LANL2DZdp(I)
No imaginary frequency
Zero-point correction: 0.194194
Enthalpy correction: 0.212008
Free energy correction: 0.145603

**DIB·BF$_3$ complex**

| | | | |
|---|---|---|---|
| C | 0.165145 | 0.191436 | 0.847807 |
| C | -0.004296 | -0.533608 | 3.498355 |
| C | 0.171285 | -1.163276 | 1.170234 |
| C | 0.073775 | 1.195753 | 1.808453 |
| C | -0.012174 | 0.816767 | 3.147828 |
| C | 0.088535 | -1.519554 | 2.515867 |
| H | 0.239389 | -1.922408 | 0.397883 |
| H | 0.068853 | 2.243195 | 1.525796 |
| H | -0.084283 | 1.581309 | 3.914691 |
| H | 0.093742 | -2.569440 | 2.790465 |
| H | -0.070887 | -0.819150 | 4.543514 |
| I | 0.295308 | 0.737721 | -1.161317 |
| O | 2.338291 | 0.598410 | -0.864872 |
| O | -1.955106 | 0.738904 | -0.920674 |
| C | -2.650005 | 1.193526 | -1.867729 |
| C | 3.020274 | 0.857104 | -1.978349 |
| C | 4.507662 | 0.790995 | -1.795174 |
| H | 4.785004 | -0.180103 | -1.378605 |
| H | 4.815272 | 1.563619 | -1.085410 |
| H | 5.001134 | 0.947188 | -2.753462 |
| C | -4.132282 | 1.266388 | -1.707395 |
| H | -4.633377 | 0.988109 | -2.635207 |
| H | -4.394116 | 2.303465 | -1.474725 |
| H | -4.441010 | 0.621531 | -0.886398 |
| O | 2.448534 | 1.124533 | -3.022300 |
| O | -2.048447 | 1.598991 | -2.926140 |
| B | -2.730461 | 2.191318 | -4.155597 |
| F | -1.696955 | 2.579619 | -4.988566 |
| F | -3.518270 | 1.204654 | -4.739715 |
| F | -3.503132 | 3.274602 | -3.750858 |

SCF energy: -1024.166167820
opt@M06-2X(SMD,CHCl3)/6-31+G(d,p)+LANL2DZdp(I)
No imaginary frequency
Zero-point correction: 0.208790
Enthalpy correction: 0.230711
Free energy correction: 0.154224

**HAIB - PhI(OH)OAc**

| | | | |
|---|---|---|---|
| H | -1.312643 | -0.613526 | 3.988140 |
| H | -1.038675 | -1.556612 | 1.707929 |
| C | -0.605080 | -0.160994 | 3.300462 |
| C | -0.449900 | -0.701498 | 2.023272 |
| O | -1.356792 | -1.131242 | -0.836755 |
| H | 0.020420 | 1.363682 | 4.685717 |
| C | 0.145438 | 0.947765 | 3.690830 |
| C | 0.467721 | -0.111643 | 1.159587 |
| I | 0.652060 | -0.920944 | -0.777935 |
| C | 1.055900 | 1.524476 | 2.805109 |
| C | 1.224826 | 0.997782 | 1.524006 |
| H | 1.637037 | 2.391230 | 3.104051 |
| H | 1.935654 | 1.438587 | 0.834097 |
| O | 2.773184 | -0.643867 | -0.306344 |
| O | 3.116184 | -1.451397 | -2.355801 |
| C | 3.550810 | -1.010349 | -1.292554 |
| H | 5.301725 | -1.486587 | -0.165897 |
| C | 5.024173 | -0.851053 | -1.011397 |
| H | 5.236641 | 0.184317 | -0.732694 |
| H | 5.607335 | -1.128876 | -1.889187 |
| H | -1.784871 | -0.287867 | -1.051919 |

SCF energy: -547.036999382
opt@M06-2X(SMD,CHCl3)/6-31+G(d,p)+LANL2DZdp(I)
No imaginary frequency
Zero-point correction: 0.155990
Enthalpy correction: 0.170298
Free energy correction: 0.113332

**HTFIB - PhI(OH)O$_2$CCF$_3$**

| | | | |
|---|---|---|---|
| H | -1.249119 | -1.144748 | 3.956307 |
| H | -0.951891 | -1.867567 | 1.600609 |
| C | -0.611002 | -0.568652 | 3.293787 |
| C | -0.445200 | -0.982440 | 1.971619 |
| O | -1.405161 | -0.878870 | -1.056617 |
| H | -0.087970 | 0.884461 | 4.792093 |
| C | 0.044195 | 0.570442 | 3.761445 |
| C | 0.380922 | -0.227645 | 1.144940 |
| I | 0.577303 | -0.819887 | -0.858873 |
| C | 0.869267 | 1.307677 | 2.911654 |
| C | 1.042982 | 0.914309 | 1.584725 |
| H | 1.376683 | 2.195777 | 3.274805 |
| O | 2.755855 | -0.759760 | -0.247504 |
| H | 1.683272 | 1.481982 | 0.917716 |
| C | 3.558543 | -1.000506 | -1.216649 |
| O | 3.293297 | -1.242835 | -2.382051 |
| F | 5.269582 | -1.914697 | 0.165956 |
| C | 5.037175 | -0.966893 | -0.756238 |
| F | 5.879891 | -1.169645 | -1.770354 |
| F | 5.342483 | 0.216627 | -0.200632 |
| H | -1.759954 | 0.014523 | -1.195318 |

SCF energy: -844.679122803
opt@M06-2X(SMD,CHCl3)/6-31+G(d,p)+LANL2DZdp(I)
No imaginary frequency
Zero-point correction: 0.133132
Enthalpy correction: 0.149377
Free energy correction: 0.086066

```
---------------------------------------------
HTIB - PhI(OH)OTs
---------------------------------------------
   H     0.869667     3.066453    -2.254657
   H    -0.178048     3.789253    -0.121635
   C     0.996207     2.472548    -1.354918
   C     0.410209     2.877437    -0.154854
   H    -2.557958     0.961132    -0.742944
   H     2.207554     0.976852    -2.339135
   C     1.754025     1.302691    -1.409130
   H    -1.109039    -0.293206    -2.316613
   H    -2.934211     1.494343     1.757729
   C    -2.029754     0.074338    -0.401396
   C     0.574851     2.115239     1.001445
   H    -4.126818     0.198425     1.650720
   C    -1.218705    -0.623522    -1.287472
   H     0.112131     2.424738     1.933633
   C    -3.075082     0.414828     1.868095
   C     1.899225     0.561420    -0.240738
   C    -2.175443    -0.344723     0.930253
   C     1.324973     0.938570     0.968096
   H    -2.883062     0.142577     2.908675
   O    -0.088466    -2.696759    -3.258333
   O     1.770340    -1.650233    -2.070612
   C    -0.540108    -1.756521    -0.835018
   S     0.558153    -2.603767    -1.949637
   H     1.455256     0.338767     1.863637
   C    -1.482400    -1.481109     1.355827
   I     3.053303    -1.196959    -0.277957
   C    -0.661082    -2.192808     0.479731
   H    -1.583332    -1.816569     2.384548
   O     0.962793    -3.855620    -1.302498
   H    -0.124611    -3.076517     0.810800
   O     4.058742    -0.591190     1.329878
   H     4.760690     0.029894     1.074480
---------------------------------------------
SCF energy: -1213.205820580
opt@M06-2X(SMD,CHCl3)/6-31+G(d,p)+LANL2DZdp(I)
    No imaginary frequency
    Zero-point correction:       0.237756
    Enthalpy correction:         0.257942
    Free energy correction:      0.187814
---------------------------------------------
```

```
---------------------------------------------
Int1 & Int2
---------------------------------------------
   C     1.189111     1.194549     2.265985
   C     0.528630     1.093810     4.948167
   C     1.152356    -0.043627     2.904472
   C     0.903033     2.383034     2.934268
   C     0.568723     2.321512     4.287490
   C     0.819117    -0.083625     4.258764
   H     1.372057    -0.957429     2.361989
   H     0.932944     3.334774     2.414377
   H     0.340155     3.238655     4.821333
   H     0.785008    -1.040395     4.770346
   H     0.268447     1.054046     6.001228
   I     1.701989     1.274116     0.231502
   O    -1.480820     1.335764     0.206128
   C    -1.748415     1.260472    -0.984612
   C    -3.142056     0.955794    -1.472381
   H    -3.424786     1.614676    -2.295446
   H    -3.841270     1.060111    -0.643530
   H    -3.170110    -0.072361    -1.844305
   C     4.938668     0.763612    -1.153215
   C     5.313104     0.388840    -3.852519
   C     4.960203    -0.538899    -1.647083
   C     5.093210     1.885217    -1.964591
   C     5.283172     1.682570    -3.331205
   C     5.154639    -0.716571    -3.015992
   H     4.827443    -1.390769    -0.988284
   H     5.063146     2.887047    -1.549024
   H     5.403195     2.539911    -3.985337
   H     5.174722    -1.721219    -3.425487
   H     5.455732     0.241199    -4.918256
   I     4.638263     1.046334     0.895368
   O     6.720468     0.806119     1.038381
   C     7.129149     0.906840     2.297137
   O     6.344911     1.116384     3.209786
   C     8.611834     0.727404     2.457957
   H     8.890254     0.909592     3.495087
   H     9.141296     1.414524     1.794265
   H     8.880518    -0.293392     2.172746
   O    -0.789414     1.426977    -1.878310
   B    -0.933764     1.384489    -3.346024
   F    -1.737148     2.446177    -3.796335
   F    -1.493298     0.165097    -3.762157
   F     0.360238     1.515244    -3.868731
---------------------------------------------
SCF energy: -1267.050032440
opt@M06-2X(SMD,CHCl3)/6-31+G(d,p)+LANL2DZdp(I)
    No imaginary frequency
    Zero-point correction:       0.299215
    Enthalpy correction:         0.329674
    Free energy correction:      0.228213
---------------------------------------------
```

**$Me_2O$**

| | | | |
|---|---|---|---|
| C | -0.011867 | 0.021475 | 1.131469 |
| H | 1.001533 | 0.055971 | 1.556880 |
| H | -0.543130 | -0.841830 | 1.557384 |
| H | -0.543620 | 0.936136 | 1.401715 |
| O | 0.030251 | -0.049860 | -0.276319 |
| C | 0.698006 | -1.209901 | -0.719946 |
| H | 1.738455 | -1.228921 | -0.364709 |
| H | 0.693631 | -1.198407 | -1.811853 |
| H | 0.190142 | -2.119464 | -0.368022 |

SCF energy: -154.958069076
opt@M06-2X(SMD,CHCl3)/6-31+G(d,p)
No imaginary frequency
Zero-point correction: 0.080494
Enthalpy correction: 0.085699
Free energy correction: 0.055171

***o*-I-BzOH**

| | | | |
|---|---|---|---|
| C | -0.020342 | 0.009518 | 1.025684 |
| C | 0.058905 | -0.043643 | 3.826325 |
| C | 0.060379 | -1.220593 | 1.698310 |
| C | -0.065246 | 1.197510 | 1.758594 |
| C | -0.017198 | 1.171761 | 3.150199 |
| C | 0.088893 | -1.227962 | 3.101029 |
| H | -0.141106 | 2.146006 | 1.238111 |
| H | -0.046118 | 2.106978 | 3.700760 |
| H | 0.147246 | -2.180534 | 3.615895 |
| H | 0.092689 | -0.070975 | 4.910388 |
| C | 0.163155 | -2.532420 | 0.992497 |
| O | 0.605170 | -2.700710 | -0.121420 |
| O | -0.273610 | -3.552190 | 1.749024 |
| H | -0.138452 | -4.375496 | 1.249017 |
| I | -0.167720 | 0.213398 | -1.063223 |

SCF energy: -431.414118511
opt@M06-2X(SMD,CHCl3)/6-31+G(d,p)+LANL2DZdp(I)
No imaginary frequency
Zero-point correction: 0.105914
Enthalpy correction: 0.115564
Free energy correction: 0.069565

***p*-bromo DIB (3b) - *p*-Br-$C_6H_4I(OAc)_2$**

| | | | |
|---|---|---|---|
| C | 0.999820 | 0.989113 | 1.904659 |
| C | 1.003706 | 0.963415 | 4.650570 |
| C | 1.019328 | -0.233916 | 2.568849 |
| C | 0.982443 | 2.199598 | 2.591653 |
| C | 0.983188 | 2.185332 | 3.984803 |
| C | 1.022489 | -0.245798 | 3.962060 |
| H | 1.033283 | -1.170131 | 2.020862 |
| H | 0.967068 | 3.145619 | 2.060959 |
| H | 0.969173 | 3.117887 | 4.537723 |
| H | 1.038073 | -1.188559 | 4.497373 |
| I | 0.997782 | 1.007458 | -0.184076 |
| O | -1.106147 | 1.188498 | 0.131639 |
| C | -1.766352 | 1.231092 | -1.007739 |
| O | -1.195017 | 1.179500 | -2.090959 |
| C | -3.258335 | 1.340160 | -0.847623 |
| H | -3.731433 | 1.425158 | -1.825245 |
| H | -3.499977 | 2.211248 | -0.233685 |
| H | -3.630380 | 0.452478 | -0.328869 |
| O | 3.102305 | 0.823785 | 0.126267 |
| C | 3.762642 | 0.805209 | -1.013693 |
| O | 3.191187 | 0.872644 | -2.095947 |
| C | 5.255363 | 0.703236 | -0.855345 |
| H | 5.621349 | 1.584531 | -0.321579 |
| H | 5.728895 | 0.638227 | -1.834281 |
| H | 5.503156 | -0.176536 | -0.256469 |
| Br | 1.005892 | 0.945353 | 6.545391 |

SCF energy: -3270.529749310
opt@M06-2X(SMD,CHCl3)/6-31+G(d,p)+LANL2DZdp(I)
No imaginary frequency
Zero-point correction: 0.184433
Enthalpy correction: 0.203628
Free energy correction: 0.132630

**$p$-bromo BTI (7) - $p$-Br-$C_6H_4I(O_2CCF_3)_2$**

| | | | |
|---|---|---|---|
| C | 0.999550 | 0.984602 | 1.889668 |
| C | 1.003507 | 0.964550 | 4.627344 |
| C | 0.969152 | -0.240464 | 2.549495 |
| C | 1.032222 | 2.200122 | 2.567232 |
| C | 1.032761 | 2.185953 | 3.959577 |
| C | 0.972565 | -0.246912 | 3.941964 |
| H | 0.943365 | -1.176899 | 2.002862 |
| H | 1.056661 | 3.144176 | 2.033943 |
| H | 1.057045 | 3.119300 | 4.510540 |
| H | 0.950079 | -1.188319 | 4.479163 |
| I | 0.998078 | 0.998142 | -0.193665 |
| O | -1.120477 | 1.182038 | 0.078256 |
| C | -1.775484 | 1.207657 | -1.042223 |
| O | -1.316219 | 1.145168 | -2.161436 |
| C | -3.297893 | 1.325469 | -0.800067 |
| F | -3.963543 | 1.393164 | -1.949424 |
| F | -3.579586 | 2.421495 | -0.084789 |
| F | -3.744490 | 0.263172 | -0.117103 |
| O | 3.117068 | 0.813065 | 0.075627 |
| C | 3.771286 | 0.826075 | -1.045458 |
| O | 3.309391 | 0.913499 | -2.161916 |
| C | 5.295625 | 0.723840 | -0.808811 |
| F | 5.728959 | 1.778855 | -0.106170 |
| F | 5.959186 | 0.688084 | -1.960732 |
| F | 5.594695 | -0.381808 | -0.115874 |
| Br | 1.005997 | 0.950573 | 6.519296 |

SCF energy: -3865.802192070
opt@M06-2X(SMD,CHCl3)/6-31+G(d,p)+LANL2DZdp(I)
No imaginary frequency
Zero-point correction: 0.138968
Enthalpy correction: 0.161932
Free energy correction: 0.079820

**$Ph_2I^+$ cation**

| | | | |
|---|---|---|---|
| C | 0.007529 | 0.171444 | 1.158954 |
| C | -0.132595 | 0.471499 | 3.888190 |
| C | -0.359958 | -0.928718 | 1.928035 |
| C | 0.303551 | 1.418060 | 1.704336 |
| C | 0.232162 | 1.556647 | 3.089939 |
| C | -0.428636 | -0.763338 | 3.312147 |
| H | -0.585888 | -1.885728 | 1.470245 |
| H | 0.583165 | 2.256028 | 1.074218 |
| H | 0.460302 | 2.516667 | 3.541576 |
| H | -0.712419 | -1.605528 | 3.934929 |
| H | -0.187633 | 0.590750 | 4.965499 |
| I | 0.129990 | -0.072198 | -0.927917 |
| C | 2.226106 | -0.231416 | -1.038644 |
| C | 4.965749 | -0.416289 | -1.172078 |
| C | 2.836959 | -1.381059 | -0.545346 |
| C | 2.934410 | 0.827716 | -1.597543 |
| C | 4.324559 | 0.719775 | -1.664067 |
| C | 4.227200 | -1.460732 | -0.613795 |
| H | 2.254143 | -2.190676 | -0.118492 |
| H | 2.426761 | 1.710810 | -1.970447 |
| H | 4.900879 | 1.530627 | -2.097661 |
| H | 4.728576 | -2.344443 | -0.232627 |
| H | 6.047258 | -0.489899 | -1.224736 |

SCF energy: -474.236864204
opt@M06-2X(SMD,CHCl3)/6-31+G(d,p)+LANL2DZdp(I)
No imaginary frequency
Zero-point correction: 0.182037
Enthalpy correction: 0.194339
Free energy correction: 0.141412

**$p$-bromoiodobenzene (1b)**

| | | | |
|---|---|---|---|
| C | 0.000000 | 0.000000 | 1.062147 |
| C | 0.000000 | 0.000000 | 3.827621 |
| C | 0.000000 | -1.211796 | 1.751975 |
| C | 0.000000 | 1.211796 | 1.751975 |
| C | 0.000000 | 1.212340 | 3.146077 |
| C | 0.000000 | -1.212340 | 3.146077 |
| H | 0.000000 | -2.154884 | 1.216157 |
| H | 0.000000 | 2.154884 | 1.216157 |
| H | 0.000000 | 2.152243 | 3.687215 |
| H | 0.000000 | -2.152243 | 3.687215 |
| I | 0.000000 | 0.000000 | -1.036719 |
| Br | 0.000000 | 0.000000 | 5.725704 |

SCF energy: -2813.781859400
opt@M06-2X(SMD,CHCl3)/6-31+G(d,p)+LANL2DZdp(I)
No imaginary frequency
Zero-point correction: 0.080939
Enthalpy correction: 0.089105
Free energy correction: 0.046736

**$Ph_2IOTf$ (4)**

| | | | |
|---|---|---|---|
| C | -0.130564 | -0.358425 | 1.034026 |
| C | -0.200871 | 0.301130 | 3.713750 |
| C | -0.240939 | -1.380256 | 1.974393 |
| C | -0.057299 | 0.985056 | 1.398234 |
| C | -0.088157 | 1.308232 | 2.753817 |
| C | -0.278946 | -1.036063 | 3.326832 |
| H | -0.295347 | -2.419677 | 1.667015 |
| H | 0.024880 | 1.763026 | 0.645558 |
| H | -0.028756 | 2.349116 | 3.055643 |
| H | -0.365936 | -1.818398 | 4.074168 |
| H | -0.228534 | 0.560694 | 4.767386 |
| I | -0.078215 | -0.866659 | -1.008369 |
| C | 1.970677 | -0.440712 | -1.256689 |
| C | 4.641651 | 0.133274 | -1.576451 |
| C | 2.898368 | -1.175660 | -0.526239 |
| C | 2.329537 | 0.566284 | -2.145923 |
| C | 3.687451 | 0.847053 | -2.301347 |
| C | 4.249882 | -0.872173 | -0.692198 |
| H | 2.585068 | -1.961535 | 0.153571 |
| H | 1.579605 | 1.112448 | -2.708583 |
| H | 3.993351 | 1.627095 | -2.991104 |
| H | 4.993371 | -1.430844 | -0.132616 |
| H | 5.695680 | 0.359676 | -1.702740 |
| S | -0.835077 | -1.534111 | -4.489368 |
| O | -2.034641 | -1.705899 | -3.659920 |
| O | -0.734836 | -2.376182 | -5.680067 |
| O | 0.413516 | -1.404751 | -3.702324 |
| C | -1.034755 | 0.169548 | -5.172475 |
| F | 0.001055 | 0.498219 | -5.949372 |
| F | -1.107250 | 1.066131 | -4.180110 |
| F | -2.150012 | 0.266754 | -5.900321 |

SCF energy: -1435.644454870
opt@M06-2X(SMD,CHCl3)/6-31+G(d,p)+LANL2DZdp(I)
No imaginary frequency
Zero-point correction: 0.210849
Enthalpy correction: 0.232335
Free energy correction: 0.155624

**PhI**

| | | | |
|---|---|---|---|
| C | 0.000000 | 0.000000 | 1.073316 |
| C | 0.000000 | 0.000000 | 3.854459 |
| C | 0.000000 | -1.214517 | 1.759502 |
| C | 0.000000 | 1.214517 | 1.759502 |
| C | 0.000000 | 1.206638 | 3.155070 |
| C | 0.000000 | -1.206638 | 3.155070 |
| H | 0.000000 | -2.154123 | 1.216998 |
| H | 0.000000 | 2.154123 | 1.216998 |
| H | 0.000000 | 2.150264 | 3.692266 |
| H | 0.000000 | -2.150264 | 3.692266 |
| H | 0.000000 | 0.000000 | 4.940038 |
| I | 0.000000 | 0.000000 | -1.030285 |

SCF energy: -242.899526652
opt@M06-2X(SMD,CHCl3)/6-31+G(d,p)+LANL2DZdp(I)
No imaginary frequency
Zero-point correction: 0.090704
Enthalpy correction: 0.097495
Free energy correction: 0.059661

**PhI (radical cation)**

| | | | |
|---|---|---|---|
| C | 0.000000 | 0.000000 | 1.067120 |
| C | 0.000000 | 0.000000 | 3.832671 |
| C | 0.000000 | -1.238506 | 1.762512 |
| C | 0.000000 | 1.238506 | 1.762512 |
| C | 0.000000 | 1.224842 | 3.142421 |
| C | 0.000000 | -1.224842 | 3.142421 |
| H | 0.000000 | -2.171535 | 1.210065 |
| H | 0.000000 | 2.171535 | 1.210065 |
| H | 0.000000 | 2.158238 | 3.694445 |
| H | 0.000000 | -2.158238 | 3.694445 |
| H | 0.000000 | 0.000000 | 4.918226 |
| I | 0.000000 | 0.000000 | -0.951705 |

SCF energy: -242.658290476
opt@M06-2X(SMD,CHCl3)/6-31+G(d,p)+LANL2DZdp(I)
No imaginary frequency
Zero-point correction: 0.090400
Enthalpy correction: 0.097277
Free energy correction: 0.058643

**$PhIO_2CCF_3^+$ (TIB$^+$)**

| | | | |
|---|---|---|---|
| C | 0.990847 | 1.025815 | 2.341445 |
| C | 0.894650 | 1.040816 | 5.082385 |
| C | 0.949952 | -0.202080 | 3.012169 |
| C | 1.002332 | 2.261313 | 2.999985 |
| C | 0.947255 | 2.252738 | 4.389434 |
| C | 0.896188 | -0.178708 | 4.401374 |
| H | 0.953213 | -1.139280 | 2.466498 |
| H | 1.046097 | 3.193084 | 2.446795 |
| H | 0.948687 | 3.193176 | 4.929748 |
| H | 0.858218 | -1.113465 | 4.950213 |
| H | 0.855251 | 1.046926 | 6.167104 |
| I | 1.112336 | 1.012661 | 0.291339 |
| O | -0.891603 | 1.059786 | 0.078310 |
| C | -1.279344 | 1.182875 | -1.185662 |
| O | -0.556300 | 1.256660 | -2.138916 |
| C | -2.824413 | 1.212751 | -1.264523 |
| F | -3.208660 | 1.356061 | -2.523648 |
| F | -3.300856 | 2.228640 | -0.544179 |
| F | -3.327451 | 0.076933 | -0.780370 |

SCF energy: -768.666731756
opt@M06-2X(SMD,CHCl3)/6-31+G(d,p)+LANL2DZdp(I)
No imaginary frequency
Zero-point correction: 0.120018
Enthalpy correction: 0.134078
Free energy correction: 0.076622

**PhIOAc (AIB·)**

| | | | |
|---|---|---|---|
| C | 1.136817 | 1.043543 | 2.344018 |
| C | 0.865242 | 1.034068 | 5.102794 |
| C | 1.026408 | -0.176375 | 3.013882 |
| C | 1.111499 | 2.258909 | 3.030840 |
| C | 0.974841 | 2.244984 | 4.418393 |
| C | 0.890376 | -0.171933 | 4.401665 |
| H | 1.044162 | -1.112096 | 2.464902 |
| H | 1.194776 | 3.198512 | 2.494673 |
| H | 0.953293 | 3.184636 | 4.961488 |
| H | 0.802775 | -1.115135 | 4.931891 |
| H | 0.758811 | 1.030331 | 6.183089 |
| I | 1.332896 | 1.053130 | 0.268174 |
| O | -1.140928 | 1.123041 | 0.171564 |
| C | -1.438696 | 1.175887 | -1.088340 |
| O | -0.618007 | 1.188020 | -2.008677 |
| C | -2.935496 | 1.204257 | -1.351063 |
| H | -3.118822 | 1.406035 | -2.406454 |
| H | -3.414023 | 1.964325 | -0.730103 |
| H | -3.359525 | 0.232561 | -1.083235 |

SCF energy: -471.238787408
opt@M06-2X(SMD,CHCl3)/6-31+G(d,p)+LANL2DZdp(I)
No imaginary frequency
Zero-point correction: 0.141193
Enthalpy correction: 0.154082
Free energy correction: 0.097722

**$PhIOAc^+$ (AIB$^+$)**

| | | | |
|---|---|---|---|
| C | 0.975225 | 1.043405 | 2.324947 |
| C | 0.894341 | 1.023095 | 5.071827 |
| C | 0.945809 | -0.189612 | 2.982288 |
| C | 0.980562 | 2.267047 | 3.001331 |
| C | 0.935804 | 2.242135 | 4.392435 |
| C | 0.899421 | -0.185756 | 4.373613 |
| H | 0.951144 | -1.121335 | 2.426952 |
| H | 1.014045 | 3.206614 | 2.460653 |
| H | 0.935158 | 3.176673 | 4.943266 |
| H | 0.869285 | -1.128546 | 4.909416 |
| H | 0.860664 | 1.015121 | 6.156754 |
| I | 1.048317 | 1.057784 | 0.257722 |
| O | -0.945881 | 1.062680 | 0.144402 |
| C | -1.349442 | 1.175089 | -1.143650 |
| O | -0.525845 | 1.240890 | -2.029543 |
| C | -2.837558 | 1.210044 | -1.271520 |
| H | -3.099954 | 1.211096 | -2.328105 |
| H | -3.213298 | 2.115350 | -0.787492 |
| H | -3.271397 | 0.344926 | -0.765795 |

SCF energy: -471.045079105
opt@M06-2X(SMD,CHCl3)/6-31+G(d,p)+LANL2DZdp(I)
No imaginary frequency
Zero-point correction: 0.143015
Enthalpy correction: 0.155017
Free energy correction: 0.103953

**PhIOAc$^+$·PhI (AIB$^+$·PhI)**

| | | | |
|---|---|---|---|
| C | 2.445633 | 1.456077 | 0.885030 |
| C | 2.646306 | 2.137755 | 3.541113 |
| C | 2.603754 | 0.436681 | 1.822480 |
| C | 2.398485 | 2.806131 | 1.230175 |
| C | 2.496369 | 3.138620 | 2.580181 |
| C | 2.702803 | 0.794773 | 3.166278 |
| H | 2.644590 | -0.604035 | 1.518211 |
| H | 2.284243 | 3.575907 | 0.474069 |
| H | 2.460028 | 4.182332 | 2.875462 |
| H | 2.820728 | 0.020215 | 3.917150 |
| H | 2.724303 | 2.406724 | 4.589957 |
| O | 0.215006 | 0.997236 | -0.814557 |
| C | -0.438792 | 0.706679 | -1.942553 |
| O | 0.166858 | 0.465698 | -2.971825 |
| C | -1.928576 | 0.698639 | -1.777919 |
| H | -2.398094 | 0.599666 | -2.755539 |
| H | -2.256199 | 1.614789 | -1.283030 |
| H | -2.205977 | -0.148738 | -1.144553 |
| I | 2.253627 | 0.943523 | -1.125307 |
| I | 5.405702 | 0.931419 | -1.236988 |
| C | 5.667209 | 2.418021 | 0.225489 |
| C | 5.957926 | 4.363122 | 2.170029 |
| C | 5.583488 | 3.759257 | -0.144876 |
| C | 5.893960 | 2.025697 | 1.543253 |
| C | 6.041318 | 3.014517 | 2.516558 |
| C | 5.731074 | 4.732975 | 0.844165 |
| H | 5.408383 | 4.042709 | -1.177628 |
| H | 5.952717 | 0.975354 | 1.809824 |
| H | 6.214926 | 2.723401 | 3.548136 |
| H | 5.668898 | 5.782049 | 0.571729 |
| H | 6.069406 | 5.126506 | 2.933787 |

SCF energy: -713.964865260
opt@M06-2X(SMD,CHCl3)/6-31+G(d,p)+LANL2DZdp(I)
No imaginary frequency
Zero-point correction: 0.234734
Enthalpy correction: 0.254526
Free energy correction: 0.183736

**PhIOH$^+$ (HIB$^+$)**

| | | | |
|---|---|---|---|
| I | -1.350792 | 0.022256 | -0.320157 |
| C | 0.713545 | 0.019162 | -0.134780 |
| C | 3.445464 | 0.009397 | 0.178943 |
| C | 1.367699 | -1.209094 | -0.017476 |
| C | 1.387222 | 1.242540 | -0.110133 |
| C | 2.770591 | 1.224405 | 0.049164 |
| C | 2.750552 | -1.200808 | 0.145368 |
| H | 0.815684 | -2.142461 | -0.047205 |
| H | 0.851129 | 2.180328 | -0.209380 |
| H | 3.317915 | 2.160783 | 0.074153 |
| H | 3.282239 | -2.141298 | 0.244677 |
| H | 4.523360 | 0.005459 | 0.306378 |
| O | -1.774723 | -0.105745 | 1.565270 |
| H | -1.807446 | 0.792068 | 1.948685 |

SCF energy: -318.442114761
opt@M06-2X(SMD,CHCl3)/6-31+G(d,p)+LANL2DZdp(I)
No imaginary frequency
Zero-point correction: 0.104583
Enthalpy correction: 0.113392
Free energy correction: 0.070142

**PhIOtBu$^+$ (tBIB$^+$)**

| | | | |
|---|---|---|---|
| C | -0.094771 | 0.481708 | 0.512813 |
| C | -0.034829 | -0.815316 | 2.937136 |
| C | 0.295190 | -0.857668 | 0.542951 |
| C | -0.448367 | 1.194363 | 1.659238 |
| C | -0.423192 | 0.523981 | 2.880449 |
| C | 0.325398 | -1.503112 | 1.776977 |
| H | 0.565846 | -1.383943 | -0.366306 |
| H | -0.737662 | 2.238737 | 1.601632 |
| H | -0.698766 | 1.053920 | 3.786299 |
| H | 0.628158 | -2.543764 | 1.828468 |
| H | -0.010405 | -1.327279 | 3.893862 |
| I | -0.136428 | 1.475011 | -1.313822 |
| O | -2.059140 | 1.534300 | -1.550434 |
| C | -2.755163 | 0.510153 | -2.353866 |
| C | -2.298031 | 0.604949 | -3.800564 |
| H | -1.242604 | 0.327346 | -3.903078 |
| H | -2.440876 | 1.619687 | -4.183267 |
| H | -2.881886 | -0.084994 | -4.416466 |
| C | -2.527920 | -0.871741 | -1.763445 |
| H | -1.485522 | -1.190433 | -1.877551 |
| H | -3.153636 | -1.597257 | -2.291125 |
| H | -2.792117 | -0.888960 | -0.701715 |
| C | -4.207108 | 0.949223 | -2.195757 |
| H | -4.345999 | 1.966508 | -2.570999 |
| H | -4.509615 | 0.909209 | -1.146088 |
| H | -4.846570 | 0.274017 | -2.771896 |

SCF energy: -475.631150576
opt@M06-2X(SMD,CHCl3)/6-31+G(d,p)+LANL2DZdp(I)
No imaginary frequency
Zero-point correction: 0.217950
Enthalpy correction: 0.231873
Free energy correction: 0.177278

**tBTFIB**

| | | | |
|---|---|---|---|
| C | -0.111293 | -0.222614 | 0.224861 |
| C | -0.481728 | 0.608272 | 2.826839 |
| C | -0.971701 | -0.948886 | 1.041032 |
| C | 0.569334 | 0.906672 | 0.667277 |
| C | 0.377962 | 1.316997 | 1.986773 |
| C | -1.153963 | -0.519507 | 2.356334 |
| H | -1.498156 | -1.820039 | 0.664527 |
| H | 1.232772 | 1.455412 | 0.006919 |
| H | 0.898866 | 2.196472 | 2.351973 |
| H | -1.819769 | -1.073571 | 3.010465 |
| H | -0.627407 | 0.935771 | 3.851470 |
| I | 0.075868 | -0.831245 | -1.778685 |
| O | -1.895461 | -0.984424 | -1.938966 |
| O | 2.288405 | -0.713896 | -1.175918 |
| C | 3.076274 | -0.955747 | -2.152956 |
| O | 2.798530 | -1.202470 | -3.316389 |
| C | 4.562805 | -0.925288 | -1.716041 |
| F | 4.876219 | 0.247397 | -1.141271 |
| F | 5.392301 | -1.106447 | -2.745874 |
| F | 4.812708 | -1.890936 | -0.816082 |
| C | -2.710032 | 0.121442 | -2.406571 |
| C | -2.550766 | 1.342411 | -1.504158 |
| H | -1.538407 | 1.758411 | -1.563576 |
| H | -2.764389 | 1.085514 | -0.461507 |
| H | -3.247714 | 2.125334 | -1.818931 |
| C | -2.352250 | 0.453717 | -3.852359 |
| H | -1.331097 | 0.843085 | -3.931051 |
| H | -3.030580 | 1.217735 | -4.244529 |
| H | -2.436489 | -0.440091 | -4.478386 |
| C | -4.131062 | -0.426891 | -2.317326 |
| H | -4.372818 | -0.687053 | -1.282309 |
| H | -4.232319 | -1.322473 | -2.937542 |
| H | -4.846761 | 0.323908 | -2.666435 |

SCF energy: -1001.862830000
opt@M06-2X(SMD,CHCl3)/6-31+G(d,p)+LANL2DZdp(I)
No imaginary frequency
Zero-point correction: 0.245988
Enthalpy correction: 0.267550
Free energy correction: 0.192784

***t*-BuOH**

| | | | |
|---|---|---|---|
| O | -2.165596 | 0.143858 | -1.161912 |
| C | -2.826221 | 0.205121 | -2.433037 |
| C | -2.345529 | 1.436639 | -3.198492 |
| H | -1.268239 | 1.373979 | -3.392810 |
| H | -2.540090 | 2.344185 | -2.618016 |
| H | -2.858321 | 1.522356 | -4.161853 |
| C | -2.532864 | -1.069889 | -3.221409 |
| H | -1.458600 | -1.160184 | -3.421224 |
| H | -3.056653 | -1.064370 | -4.182643 |
| H | -2.853675 | -1.949707 | -2.654531 |
| C | -4.310166 | 0.314502 | -2.111548 |
| H | -4.503377 | 1.211333 | -1.514474 |
| H | -4.642453 | -0.559706 | -1.542685 |
| H | -4.899329 | 0.375678 | -3.031446 |
| H | -1.212917 | 0.071575 | -1.317949 |

SCF energy: -233.582747961
opt@M06-2X(SMD,CHCl3)/6-31+G(d,p)
No imaginary frequency
Zero-point correction: 0.136174
Enthalpy correction: 0.143788
Free energy correction: 0.107255

**$TfO^-$ anion**

| | | | |
|---|---|---|---|
| S | 1.964595 | 0.873785 | 1.157607 |
| O | 2.145624 | -0.560874 | 0.897388 |
| O | 2.572884 | 1.774646 | 0.169210 |
| O | 2.118943 | 1.282486 | 2.560439 |
| C | 0.155194 | 1.111353 | 0.862314 |
| F | -0.203308 | 2.389319 | 1.042646 |
| F | -0.574407 | 0.361679 | 1.699021 |
| F | -0.179525 | 0.767605 | -0.388625 |

SCF energy: -961.386451911
opt@M06-2X(SMD,CHCl3)/6-31+G(d,p)
No imaginary frequency
Zero-point correction: 0.027893
Enthalpy correction: 0.035875
Free energy correction: -0.004287

**TsO⁻ anion**

| | | | |
|---|---|---|---|
| H | -0.042568 | -0.011778 | 2.144158 |
| C | -0.034775 | 0.538565 | 1.207956 |
| C | -0.031924 | 1.932382 | -1.202339 |
| C | -0.038351 | -0.155652 | 0.000000 |
| C | -0.031924 | 1.932382 | 1.202339 |
| C | -0.031121 | 2.649598 | 0.000000 |
| C | -0.034775 | 0.538565 | -1.207956 |
| H | -0.031067 | 2.472412 | 2.146230 |
| H | -0.042568 | -0.011778 | -2.144158 |
| H | -0.031067 | 2.472412 | -2.146230 |
| C | -0.064201 | 4.156092 | 0.000000 |
| H | -1.097771 | 4.520205 | 0.000000 |
| H | 0.428854 | 4.562180 | -0.887321 |
| H | 0.428854 | 4.562180 | 0.887321 |
| S | 0.040225 | -1.950829 | 0.000000 |
| O | -0.650157 | -2.373347 | -1.241111 |
| O | 1.487499 | -2.279161 | 0.000000 |
| O | -0.650157 | -2.373347 | 1.241111 |

SCF energy: -894.712862928
opt@M06-2X(SMD,CHCl3)/6-31+G(d,p)
No imaginary frequency
Zero-point correction: 0.131644
Enthalpy correction: 0.141746
Free energy correction: 0.095947

**TsOH**

| | | | |
|---|---|---|---|
| H | -0.011908 | 2.145085 | -0.195887 |
| C | -0.561412 | 1.212187 | -0.118377 |
| C | -1.954692 | -1.204830 | 0.048516 |
| C | 0.111950 | -0.005271 | -0.084480 |
| C | -1.953702 | 1.204880 | -0.066705 |
| C | -2.666400 | 0.004150 | 0.018541 |
| C | -0.566869 | -1.221640 | -0.002228 |
| H | -2.493040 | 2.147115 | -0.098940 |
| H | -0.019299 | -2.158733 | 0.015376 |
| H | -2.498185 | -2.143876 | 0.109525 |
| C | -4.169839 | -0.004493 | 0.071899 |
| H | -4.521032 | -0.528089 | 0.966575 |
| H | -4.583469 | -0.527699 | -0.796135 |
| H | -4.570981 | 1.011102 | 0.085635 |
| S | 1.878215 | -0.014387 | -0.111207 |
| O | 2.356766 | 1.256880 | -0.630406 |
| O | 2.350737 | -1.267358 | -0.665869 |
| O | 2.286169 | -0.094928 | 1.448037 |
| H | 2.239171 | 0.789809 | 1.859732 |

SCF energy: -895.160713018
opt@M06-2X(SMD,CHCl3)/6-31+G(d,p)
No imaginary frequency
Zero-point correction: 0.143426
Enthalpy correction: 0.154896
Free energy correction: 0.106391

**$H_2O$**

| | | | |
|---|---|---|---|
| H | 0.000000 | 0.768335 | -0.470418 |
| O | 0.000000 | 0.000000 | 0.114264 |
| H | 0.000000 | -0.768335 | -0.470418 |

SCF energy: -76.403034277
opt@M06-2X(SMD,CHCl3)/6-31+G(d,p)
No imaginary frequency
Zero-point correction: 0.021285
Enthalpy correction: 0.025065
Free energy correction: 0.003634
